# Adductor Insertion Avulsion Syndrome with Proximal Femoral Shaft Stress Fracture: Not Only Found in Young Athletes

**DOI:** 10.5334/jbsr.2014

**Published:** 2020-05-06

**Authors:** Anatole Pauchet, Ana Falticeanu, Olivier Lebecque

**Affiliations:** 1 Université catholique de Louvain, CHU UCL Namur, Department of Radiology, Yvoir, BE

**Keywords:** adductor insertion avulsion syndrome, stress fracture, thigh splints

## Abstract

The adductor insertion avulsion syndrome, also called “thigh splints,” is usually considered a sports injury, causing thigh and groin pain. It is related to chronic traction stress of the adductor muscles at their insertion site along the posterior margin of the proximal and mid-femoral diaphysis, and it can get complicated by stress fracture. We report the case of a 64-year-old woman—significantly older than previously reported cases—with a history of complete functional loss of the right hip following intensive physiotherapy and a final diagnosis of adductor insertion avulsion syndrome.

## 1. Introduction

The adductor insertion avulsion syndrome—also called “thigh splints”—is part of the differential diagnosis of sports injuries and hip pain [1]. It has been mostly described in young athletes and army trainees [1,2,3]. It involves a periosteal reaction of the posteromedial femoral shaft due to the repeated stress of the adductor muscle [2] and can be associated with a stress fracture in advanced cases [3]. The diagnosis is usually based on magnetic resonance imaging (MRI) of the proximal femoral region [3]. However, a whole-body bone scan is commonly performed beforehand, helping to pinpoint the abnormality at the adductor’s insertion site [2,3,4]. Patients undergo conservative treatment with rest and gradual return to physical activity [1].

This case emphasizes the importance of recognizing the imaging features of the adductor insertion avulsion syndrome, not to be confused with gluteus maximus insertion’s lesion, especially in older patients. To the best of our knowledge, no case has ever been reported in patients older than 50 years, and only two cases have been reported so far in patients older than 40 years old [3,5].

## 2. Case Report

A 64-year-old woman presented to the emergency department with acute right hip pain and near total functional loss. The patient had a history of multiple sclerosis for which intensive physiotherapy sessions have been required. She had neither bisphosphonate nor cortisone in her drug regimen. The pain was not relieved by pain killers and anti-inflammatory drugs.

**Figure 1:**
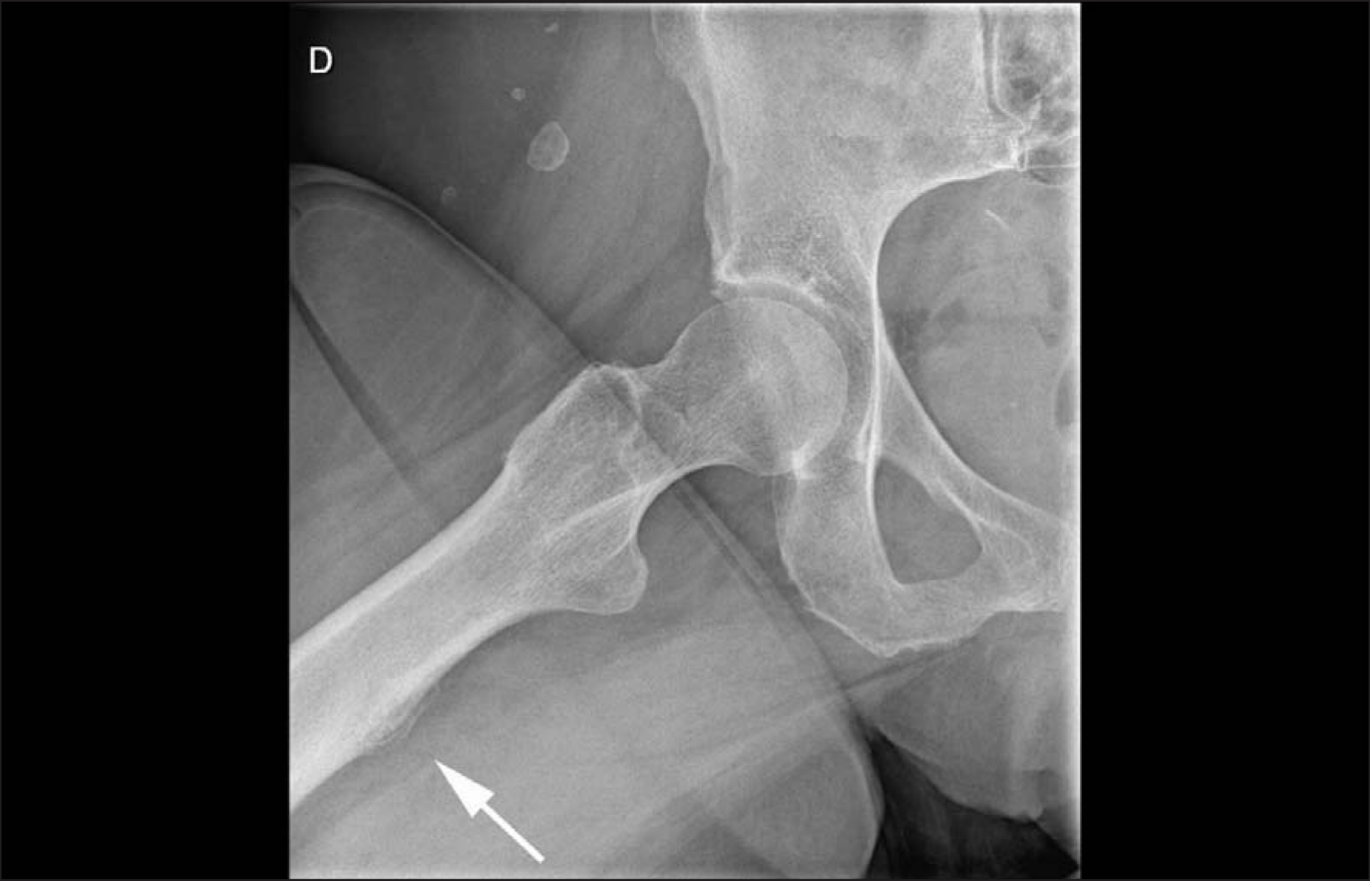
Plain radiograph of the right hip showing a mature periosteal reaction along the posteromedial proximal femoral diaphysis (arrow), without any fracture identified.

**Figure 2:**
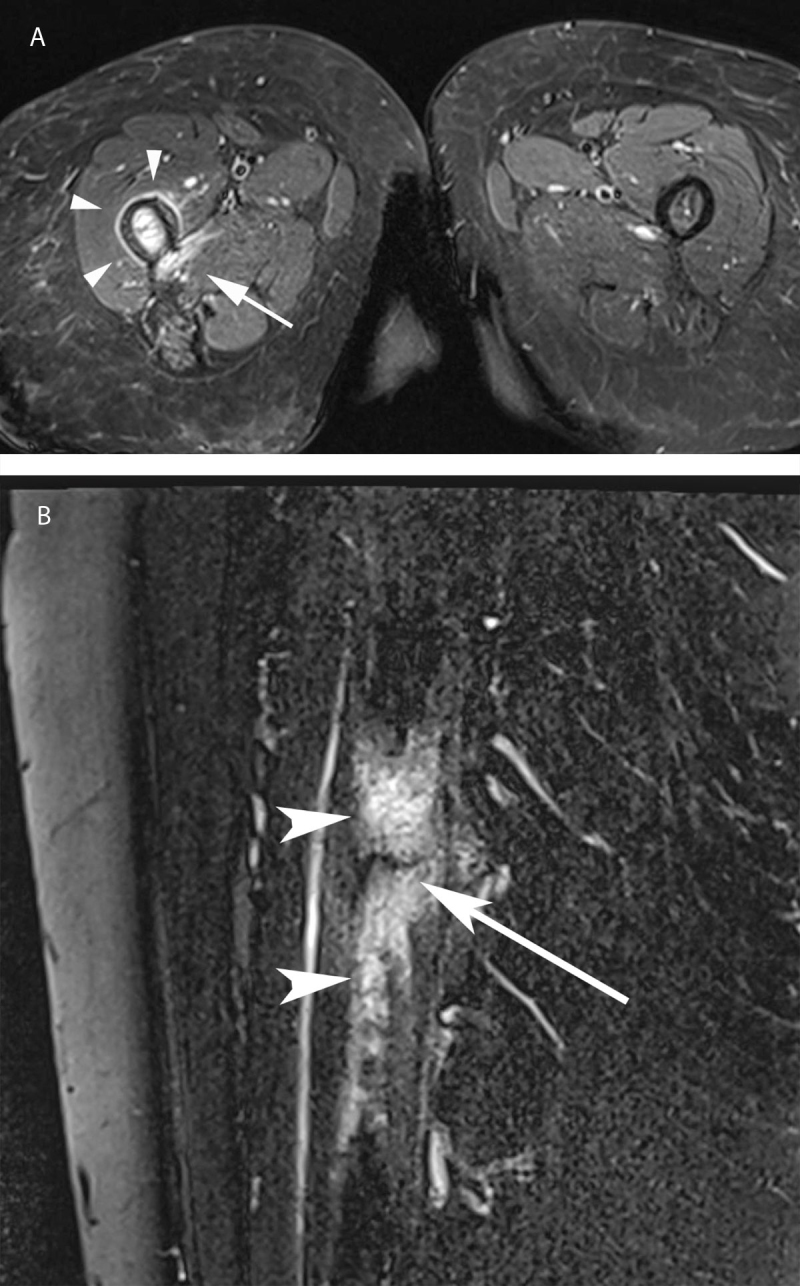
**a)** Right proximal femur MRI: axial T2-weighted inversion recovery (IR) image shows high signal intensity at the insertion site of right adductors muscles (arrow), extending around the diaphysis as a circumferential ring (arrowheads). **b)** Right proximal femur MRI: sagittal STIR image shows high signal intensity interesting the posterior soft tissue, and the bone marrow (arrowheads) with a linear low signal component interesting the medial cortical bone of the femoral shaft (arrow), consistent with stress fracture.

**Figure 3:**
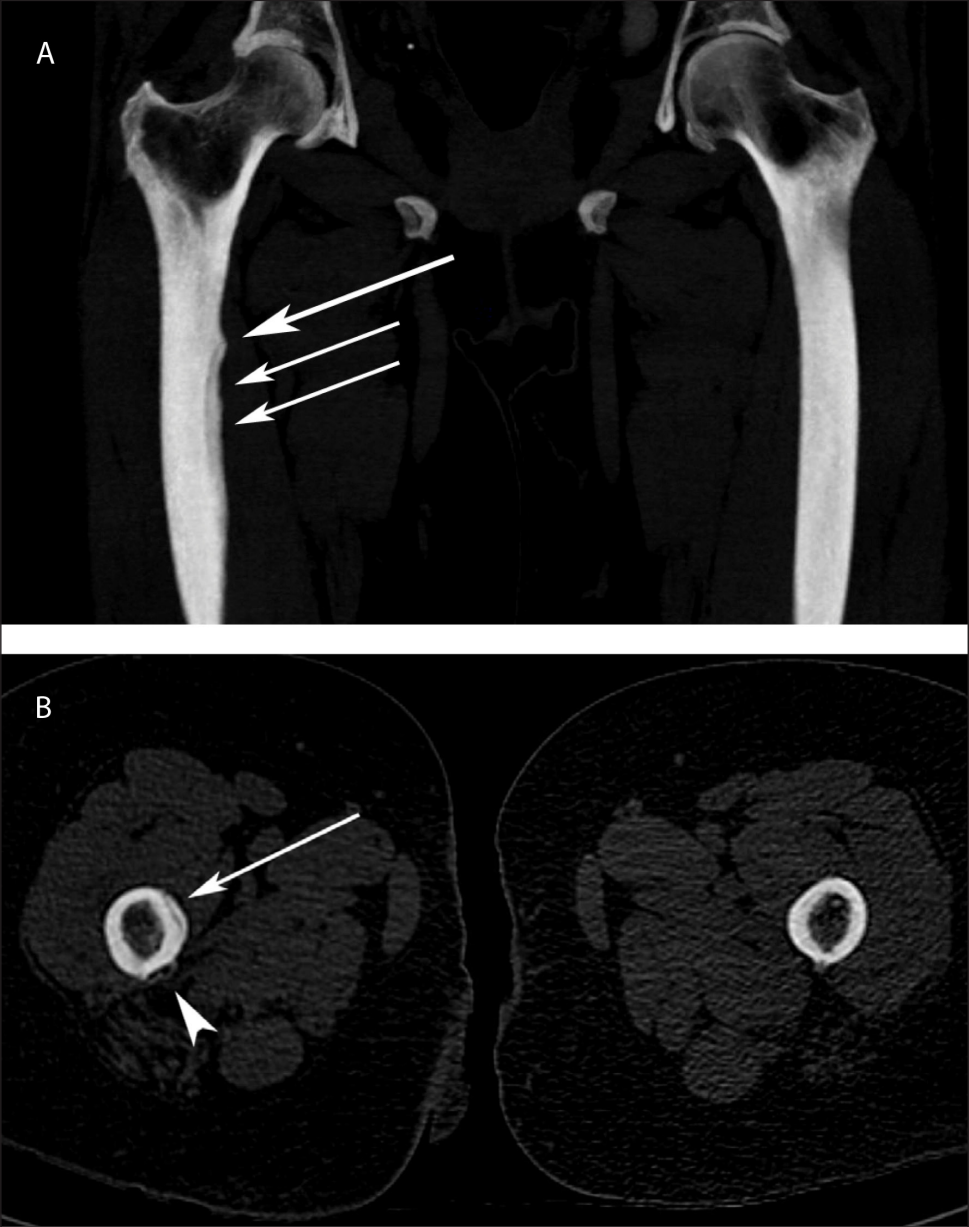
**a)** Coronal CT maximum intensity projection reconstruction of the proximal femur shows medial periosteal thickening and fine line of lucency consistent with stress fracture (arrows). **b)** Axial CT image of the proximal femur shows the medial stress fracture (arrow) with a periosteal reaction on its posteromedial part (site of adductor’s avulsion) (arrowhead).

The initial pelvis and right hip radiographs showed a periosteal reaction of the posteromedial proximal femoral shaft (Figure 1). A whole-body bone scan—performed shortly after—showed an increased radiotracer uptake at the same site on delayed images and a computed tomography exam re-affirmed the periosteal reaction. Two weeks later, the patient underwent a hip MRI in our institution. The T2-STIR sequences showed high signal intensity in the soft tissues at the insertion of the adductor’s muscles, with a circumferential rim shaped extension along the femoral shaft (Figure 2a). High T2-STIR signal intensity was found in the femoral bone marrow at the same height, adjacent to the adductor’s insertion site. Gadolinium-enhanced fat-suppressed T1-weighted images showed enhancement of the corresponding bone marrow and soft tissues at the femoral insertion of the adductor’s muscles. MRI also revealed a cortical thickening and an underlying stress fracture of the medial cortical bone of the femoral shaft, appearing as a hypointense fracture line on both T1- and T2-weighted sequences (Figure 2b), associated with cortical thickening. High-resolution computed tomography confirmed the stress fracture (Figure 3). The diagnosis of adductor insertion avulsion syndrome with stress fracture was, therefore, made two weeks after the initial admission.

## 3. Discussion

The adductor insertion avulsion syndrome is a rare cause of hip pain, with very few cases reported. The pathogenesis has been described by Charkes et al. as similar to the classical shin splints, and leading less commonly to a cortical stress fracture as a final consequence [2]. We found 23 patients diagnosed with an adductor insertion avulsion syndrome in literature [2,3,4,5,6,7,8,9,10] with a mean age of 19.2 years. Only two patients over 40 have been reported with thigh splints, and neither of them had mentioned any recent athletic activity [3,5]. We report the case of a 64-year-old patient, making it the oldest case in literature, and we suspect this pathology was caused by the intensive physiotherapy sessions.

The initial radiographs can be negative or they may show a periosteal reaction on the posteromedial femoral shaft [1,2,3,6]. Bone scan helps pinpoint this pathology, often located at the edge of the field of view of a hip MRI [3]. An increased radiotracer uptake along the posteromedial femoral diaphysis is seen on delayed images [1,2] and computed tomography can confirm the periosteal reaction and a stress fracture, if present [4]. The most common finding on MRI is a high signal intensity on T2 STIR weighted image in the soft tissue along the posteromedial proximal femoral shaft at the adductor’s insertion site [1,3,4,5,6,7,8,9,10]. In advanced cases, a high signal intensity on T2 STIR weighted images can be found in the adjacent bone marrow [1,3,4,5,6,7,8,10], which may sometimes reveal an associated stress fracture, appearing as a linear low signal component through the cortex. The MRI findings are not specific, and the differential diagnosis includes tumor and osteomyelitis, requiring an follow-ups, especially in young patients [3,6,7,11]. One of the main differential diagnosis to consider, especially in older patients, is a lesion of the maximus gluteus insertion, as it is next to the insertion site of the adductor muscles [12]. Common imaging findings may include bone scan hot spots at the femoral proximal region and periosteal reaction on radiograph and computed tomography [12,13]—findings very similar to the thigh splint.

High-resolution computed tomography may not be required for diagnosis after the MRI, especially if a bone scan has previously been performed. Two cases of ultrasound diagnosed thigh splints have been reported [9,10]. Ultrasound findings may include cortical irregularity at the adductor’s insertions site with adjacent soft tissue hypoechogenicity and hyperemia [1].

## 4. Conclusion

The adductor insertion avulsion syndrome is part of the hip pain differential diagnosis. It is caused by chronic traction stress and may be associated to a stress fracture, potentially causing complete functional loss. Although mainly encountered in young athletes, our case report highlights that elderly people can also be affected. It should not be confused with gluteus maximus insertion’s lesion, a pathology affecting mainly older patients, with similar topography of bone scan and MRI abnormalities.
